# *Eleutherococcus* Species Cultivated in Europe: A New Source of Compounds with Antiacetylcholinesterase, Antihyaluronidase, Anti-DPPH, and Cytotoxic Activities

**DOI:** 10.1155/2019/8673521

**Published:** 2019-03-10

**Authors:** Kuba Adamczyk, Marta Olech, Jagoda Abramek, Wioleta Pietrzak, Rafał Kuźniewski, Anna Bogucka-Kocka, Renata Nowak, Aneta A. Ptaszyńska, Alina Rapacka-Gackowska, Tomasz Skalski, Maciej Strzemski, Ireneusz Sowa, Magdalena Wójciak-Kosior, Marcin Feldo, Daniel Załuski

**Affiliations:** ^1^Department of Pharmacognosy, Ludwik Rydygier Collegium Medicum, Nicolaus Copernicus University, 9 Marie Curie-Skłodowska Street, 85-094 Bydgoszcz, Poland; ^2^Department of Pharmaceutical Botany, Medical University of Lublin, 1 Chodźki Street, 20-093 Lublin, Poland; ^3^Department of Biology and Genetics, Medical University of Lublin, 4a Chodźki Street, 20-093 Lublin, Poland; ^4^Department of Botany and Mycology, Institute of Biology and Biochemistry, Faculty of Biology and Biotechnology, Maria Curie-Skłodowska University, Lublin, Poland; ^5^Department of Analytical Chemistry, Medical University of Lublin, Chodźki 4a, 20-093 Lublin, Poland; ^6^Department of Vascular Surgery and Angiology, Medical University of Lublin, Lublin, Poland

## Abstract

Secondary metabolites of the roots of *Eleutherococcus* spp. cultivated in Poland, or the bioactivity, are not fully known. The 75% methanol extracts of five *Eleutherococcus* spp. (*E. senticosus*, *E. divaricatus*, *E. sessiliflorus*, *E. gracilistylus*, and *E. henryi*) were examined for the content of polyphenols and phenolic acids as well as for antiacetylcholinesterase, antihyaluronidase, anti-DPPH^∗^, and cytotoxic activities. The richest in polyphenols were the roots of *E. henryi* (10.4 mg/g DW), while in flavonoids the roots of *E. divaricatus* (6.5 mg/g DW). The richest in phenolic acids occurred the roots of *E. henryi* [protocatechuic acid (1865 *μ*g/g DE), caffeic acid (244 *μ*g/g DE), and *p*-coumaric and ferulic acids (55 *μ*g/g DE)]. The highest inhibition of AChE was observed for *E. gracilistylus* and *E. sessiliflorus* (32%), at the concentration of 100 *μ*g/0.19 mL of the reaction mixture, while that of Hyal for the roots of *E. henryi* (40.7%), at the concentration of 100 *μ*g/0.16 mL of the reaction mixture. Among five species tested, the *E. henryi* extract exhibited the strongest HL-60 cell line growth's inhibition (IC_50_ 270 *μ*g/mL). The extracts reduced DPPH^∗^ in a time-dependent mode, at the concentration of 0.8 mg/mL. After 90 min from 14.7 to 26.2%, DPPH^∗^was reduced. A phytochemical composition and activity of the *Eleutherococcus* species, cultivated in Poland, are still under research; however, on the basis of the results obtained, it may be concluded that they may become a source of phytochemicals and be useful for Europe's citizens.

## 1. Introduction

There are at least 250,000 species of higher plants that exist on the planet, but merely 5-10% of these have been investigated so far. Because plants represent an unlimited source of novel chemical entities (NCE) with potential as drug leads, they are still used by herbalists to treat various ailments [1]. The *Eleutherococcus* species are mostly found in eastern Asia and the far eastern areas of the Russian taiga. The species are a source of plant-based chemicals, such as eleutherosides (derivatives of lignans, coumarins, and phenylpropanoids). These compounds are said to be responsible for antioxidative, immunomodulating, hepatoprotective, antirheumatic, or anti-inflammatory activities [2–8]. One of the best-known worldwide species from this genus is *Eleutherococcus senticosus* (Rupr. et Maxim.) Maxim. that has been used in TCM (Traditional Chinese Medicine) for years to treat combined neurosis, coronary heart disease, inflammation, angina pectoris, stress-induced pathophysiologic changes, and menopausal syndrome. That species has been recognized as an adaptogenic plant. The adaptogen increases the state of nonspecific resistance and is safe in long-term use in the appropriate dose level. In addition to this, the adaptogen should reduce stress reactions in the alarm phase. Some researchers think that eleutherosides are responsible for that activity, whereas the others do not. Nevertheless, there has been a lack of studies on isolated eleutherosides and their adaptogenic activity [3, 5, 9–11].

In the literature, there is no information on an antihyaluronidase (anti-Hyal) activity of *Eleutherococcus* spp., apart from Załuski et al.'s reports [6, 12]. Hyaluronidase is the enzyme that takes part in tumor invasiveness and the development of inflammation. The overexpression of Hyal has an impact, among others, on the development of varicose veins [13, 14]. The Hyal inhibitors used in the treatment have a low inhibitory potency (aescin isolated from *Hippocastani semen*; *Aesculus hippocastanum* L.), and new inhibitors are required in the clinic.

In the TCM, *Eleutherococcus* spp. are used to improve a mental process; however, the biochemical mechanisms of their action are not yet known. In a majority of studies, an inhibitory effect on amyloid *β* (A*β*) peptide formation is reported, in *in vitro* and *in vivo* models [15, 16]. There are not a lot of reports on the inhibitory activity of *Eleutherococcus* spp. towards acetylcholinesterase (AChE).

Taking into account the above information, the phytochemical and ethnopharmacological knowledge of *Eleutherococcus* spp. should be now confirmed with using new approaches, in *in vitro* and *in vivo* models. A chemical metabolomics strategy is used to identify the potential biomarkers for assessing their action mechanism and searching for the structure-activity relationship. The knowledge about their valuable medicinal properties is based, mainly, on the Traditional Chinese Medicine and scientific investigations done in Asia. The European species have not been investigated in details; therefore, the results of these studies may become an alternative for the species imported from Asia, very often, with poor quality and substituted with *Periploca sepium*.

As part of a program to search for bioactive constituents from *Eleutherococcus* species, the aim of this study was to evaluate whether the 75% methanol extracts from the roots of five *Eleutherococcus* species contain phytochemicals inhibiting the activity of Hyal, AChE, DPPH^∗^, and the HL-60 cell line's growth. The phenolic acid profile has been also determined.

## 2. Experimental

### 2.1. Standards and Reagents

Standards of caffeic, ferulic, gallic, protocatechuic, 4-OH-benzoic, salicylic, rosmarinic, vanillic, syringic, *m*-coumaric, *p*-coumaric, and veratric acids and LC grade acetonitrile were provided by Sigma-Aldrich Fine Chemicals (St. Louis, MO, USA). Gentisic and sinapic acid were provided by ChromaDex (Irvine, USA). Folin-Ciocalteu reagent, 1,1-diphenyl-2-picryl-hydrazyl (DPPH), ascorbic acid, DMSO, bovine albumin, hyaluronidase from bovine testes type I-S, *Streptococcus equi* hyaluronic acid, DTNB (5,5′-dithiobis(2-nitrobenzoic acid)), acetylcholinesterase (AChE), ACTI (acetylthiocholine iodide), and sodium phosphate buffer pH 7.0 were obtained from Sigma-Aldrich. FeCl_3_ and methanol were obtained from POCH (Lublin, Poland). The acetate buffer, pH 4.5, was purchased from J.T.Baker, USA. Liquid chromatography- (LC-) grade methanol (MeOH) and acetonitrile (ACN) were purchased from Merck (Darmstadt, Germany). LC grade water was prepared using a Millipore Direct-Q3 purification system (Bedford, MA, USA). All reagents were of analytical grade.

### 2.2. Plant Materials

The roots of *E. senticosus* (Rupr. et Maxim.) Maxim., *E. divaricatus* (Siebold et Zucc.) S.Y. Hu, *E. sessiliflorus* (Rupr. et Maxim.) S.Y. Hu, *E. gracilistylus* (W.W. Smith) S.Y. Hu, and *E. henryi* Oliv. were obtained from the arboretum in Rogów (Poland) in October 2017. Voucher specimen was deposited at the Department of Pharmacognosy, Collegium Medicum in Bydgoszcz, Poland (Nr.: ES01/17, ED02/17, ESes04/17, EG05/17, EH06/17). The following are the growth's conditions: geographic data 51° 49′N and 19° 53′E; the average, long-term temperature—20.1°C, the 6b^th^ subclimate (according to USDA Frost Hardiness Zones), and the second zone according to the Kórnik's category. These plants are grown on the acidic, luvic, and sandy soils.

### 2.3. Dried Material Extraction with 75% Methanol

The air-dried, grinded roots (10 g each) were soaked in 100 mL of 75% methanol for 24 h. Subsequently, the samples were sonificated three times (100, 2 × 50 mL of 75% methanol) in the following conditions: room temperature and time 15 min for each cycle. Finally, 200 mL of each extract was obtained. The solvents were evaporated under vacuum conditions at 45°C and subjected to lyophilisation. The extraction yield was calculated based on the dry weight of the extract (%).

### 2.4. Total Phenolic Content (TPC)

The total phenolic content of extracts was determined using the method of Singleton and Rossi [17]. Gallic acid was used to calculate the calibration curve (20-100 *μ*g/mL; *y* = 0.0026*x* + 0.044; *r*^2^ = 0.999), and TPC was expressed as gallic acid equivalents (GAE/mL). The experiments were done in triplicate.

### 2.5. Total Flavonoid Content (TFC)

The TFC in investigated samples was determined using the FeCl_3_ method [18]. TFC were expressed as the mean (±S.E.) mg of quercetin equivalent (QEs/mL for FeCl_3_ method; 20-100 *μ*g/mL; *y* = 0.0041*x* + 0.236; *r*^2^ = 0.999). The experiments were done in triplicate.

### 2.6. LC-ESI-MS/MS Conditions of Analysis of Phenolic Acids

Phenolic acid profile was determined according to the modified methods of Nowacka et al., [19], Pietrzak et al., [20], and Załuski et al., [12]. In this case, the Agilent 1200 Series HPLC system (Agilent Technologies, USA) connected to a 3200 QTRAP MS/MS (AB Sciex, USA) was used. The separation's conditions were as follows: temperature 25°C, a Zorbax SB-C18 column (2.1 × 50 mm, 1.8 *μ*m particle size; Agilent Technologies. USA), and injection volume 3 *μ*L. Gradient elution was applied using water containing 0.1% HCOOH (A) and methanol (B) with the flow rate of 500 *μ*L/min. The gradient was as follows: 0-1 min 5% B; 2-3 min 20% B; 5-8 min 100% B; and 9-12 min 5% B.

To quantify phenolic acids, the parameters of analysis were optimized. The 3200 QTRAP ESI-MS/MS system in the negative mode was used. The optimal mass spectrometer parameters were as follows: curtain gas 25 psi; capillary temperature 500°C; nebulizer gas 60 psi; and negative ionization mode source voltage -4500 V. Nitrogen was used as a curtain and collision gas. For the quantitative analysis of compounds, the data were processed using Analyst 1.5 software from AB Sciex, USA, and in a multiple reaction monitoring system (MRM). The identification of analytes was done by comparing the retention times and *m*/*z* values obtained by MS and MS2 with the mass spectra of the corresponding standards tested under the same conditions. The calibration curves obtained in the MRM mode were used for the quantification of all analytes. The identified compounds were quantified based on their peak areas and comparison with a calibration curve for the corresponding standards. Linearity ranges for calibration curves were specified. The limits of detection (LOD) and quantification (LOQ) for phenolic acids were determined at a signal-to-noise ratio of 3 : 1 and 10 : 1, respectively, by injecting a series of dilute solutions of known concentrations.

### 2.7. Antihyaluronidase Studies

The modified spectrophotometric method was used to determine the antihyaluronidase (Hyal) activity of the extracts [21]. The extracts (4.6 mg/mL) were dissolved in 10% water ethanol solution, the final concentration in the reaction's mixture was 100 *μ*g/0.16 mL. 50 *μ*L of enzyme (30 U/mg) in acetate buffer pH 4.5, 50 *μ*L of sodium phosphate buffer (50 mM, pH 7.0; with 77 mM NaCl and 1 mg/mL of albumin), and 22 *μ*L of the analyzed samples were combined and next incubated at 37°C for 10 min. After that time, 50 *μ*L of HA (0.3 mg/mL of acetate buffer pH 4.5) was added and incubated at 37°C for 45 min. The unhydrolyzed HA was precipitated with 1 mL acid albumin solution (0.1% bovine serum albumin in 24 mM sodium acetate and 85 mM acetic acid). After 10 min incubation of the mixture at room temperature, the absorbance of the mixture was measured at 600 nm (Multi-Detection Microplate Reader Synergy™ HT, BioTek). Aescin was used as the positive control at the following concentration 0.05, 0.1, 0.2, 0.4, 0.6, and 0.8 mg/0.16 mL, and the absorbance in the absence of enzyme was used as the blind control. All assays were done in triplicate. The percentage of inhibition was calculated as follows:

(1) $$\begin{array}{c} \%\text{ inhibition}=\frac{\left(\text{AB}-\text{AE}\right)}{\left(\text{AS}-\text{AE}\right)}\times 100, \end{array}$$

where AB is the absorbance of the enzyme+substrate+extract, AE is the absorbance of the enzyme+substrate sample, and AS is the absorbance of the enzyme+substance sample.

### 2.8. Antiacetylcholinesterase Studies

To determine the ability of the extracts to inhibit AChE, the modified spectrophotometric method of Ellman et al. [22] was applied. The extracts (1.0 mg/mL) were dissolved in 10% water ethanol solution. The final concentration in the reaction's mixture was 100 *μ*g/0.19 mL. Physostigmine was used as the positive control at the following concentrations: 2, 3, 4, 15, 30, and 40 *μ*g/0.19 mL. Every assay was done in triplicate.

### 2.9. Cytotoxic Activity

Leukemic cells (HL-60-human Caucasian promyelocytic leukemia from American Type Culture Collection (ATCC CCL-240™)) were incubated at the concentration of 5 × 10^5^ cells/mL in 5% CO_2_ atmosphere for 24 h at 37°C, in a growing medium [RPMI 1640 medium (Sigma-Aldrich, St. Luis, USA), with 15% fetal bovine serum (Sigma-Aldrich), 2 mM L-glutamine, and antibiotics (100 U/mL penicillin, 100 *μ*M/mL streptomycin, and 2.5 *μ*g/mL amphotericin B; Gibco, Carlsbad, USA)].

The trypan blue test was used to assay the extracts' cytotoxicity. The cell lines were treated with different concentrations of the extracts (from 10 to 500 *μ*g/mL of cell culture) and incubated for 24 h at 37°C in air atmosphere humidified by 5% CO_2_. After that, the medium was removed from each plate by aspiration, the cells were washed with PBS and centrifuged at 800 rpm for 10 min, and PBS was removed by aspiration. Then, 10 *μ*L suspension cells were incubated for 5 min with the 10 *μ*L 0.4% trypan blue solution (Bio-Rad Laboratories Inc., Hercules, CA) and analyzed using an Olympus BX41 light microscope. The extracts were dissolved in DMSO. As a positive control, podophyllotoxin was used. The experiments were done in triplicate.

### 2.10. DPPH Assay

The DPPH^∗^ scavenging activity of the extracts was measured by using the modified method of Brand-Williams et al. [23]. The extracts' concentration was 0.8 mg/mL. As a positive control, ascorbic acid was used (1, 10, 20, 40, and 80 *μ*g/mL). A multidetection BioTek spectrophotometer was applied to measurement of absorbance. The experiments were done in triplicate.

### 2.11. Statistical Analysis

Determinations were performed by triplicate. The Statistica 7.0. programme (StatSoft, Cracow) was used for the statistical analysis. The evaluations were analyzed for one-factor variance analysis. Statistical differences between the treatment groups were estimated by Spearman's (*R*) and Person's (*r*) tests. All statistical tests were carried out at significance level of *α* = 0.05.

## 3. Results and Discussion

### 3.1. Extraction Yield, TPC, and TFC Contents

The extraction of the roots yielded 5.7 and 10.7%. The extracts were a yellow-brown, odorless powder. The 75% methanol root extracts contained 4.1 to 10.4 mg of polyphenols (mg GAE/g DW) and 1.8 to 6.5 mg of flavonoids (mg QEs/g DW), (Table 1). The richest in polyphenols were the roots of *E. henryi* (10.4 mg/g DW), while in flavonoids the roots of *E. divaricatus* (6.5 mg/g DW). The previous studies of Załuski et al. [24] have shown that the 75% ethanol root extracts contained 6.9 to 10.6 mg/g DW of polyphenols. Those results are in an agreement with those obtained in another study that may suggest that both 75% ethanol and 75% methanol are the good solvents for polyphenol extraction. There is not many literature reports on polyphenols in *Eleutherococcus* spp. growing, e.g., in Asia. Ondrejovič et al. [25] studied the TPC content in the roots of *E. senticosus*, the roots of which contained 6.2 to 21.8 mg/g plant material of polyphenols. The amount was dependent on the ethanol concentration and extraction's temperature. The other studies have revealed that the roots of *E. senticosus* and *E. koreanum*, growing in Korea, contained 44 and 34 mg GAE/g DW of TPC [5]. As may be seen, the TPC is dependent on species, the type of raw material, extraction type, and solvent used. In this case, it is hard to make a reliable comparison; however, this is important that *Eleutherococcus* spp. growing in Poland contain polyphenols. When comparing the *Eleutherococcus* spp. with other medicinal plants, it should be also reported that the content of polyphenols depends on a morphological part of plants. The underground and woody tissues contain less polyphenols than the aerial parts.

### 3.2. LC-ESI-MS/MS Analysis of Phenolic Acids

**Figure 1:**
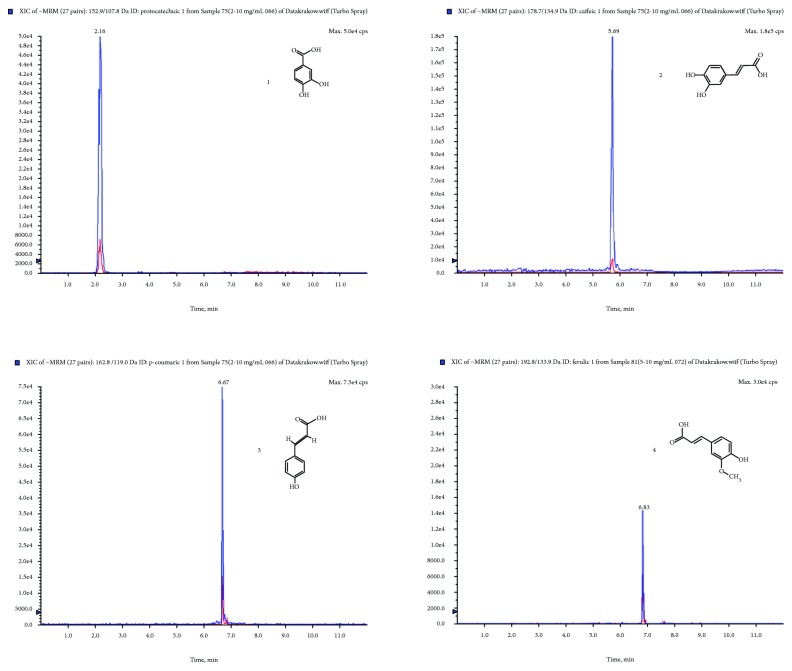
An exemplary chromatogram in the MRM mode of phenolic acids occurring in *E. henryi*: 1—protocatechuic acid; 2—caffeic acid; 3—*p*-coumaric acid; 4—ferulic acid.

To identify phenolic acids, a triple quadrupole tandem mass spectrometer (MS/MS) with multiple-reaction monitoring mode (MRM) spectra was employed (Tables 2 and 3). Among fourteen phenolic acids, just five were qualitatively and quantitatively determined in the roots (Table 4, Figure 1). The content of phenolic acids ranged between 11 and 1865 *μ*g/g DE. Among five identified acids, the richest occurred in the roots of *E. henryi* (protocatechuic acid occurred in the largest amount, 1865 *μ*g/g DE; followed by caffeic acid, 244 *μ*g/g DE; and *p*-coumaric and ferulic acids, 55 *μ*g/g DE, respectively). Protocatechuic acid has been detected in all species as a predominant constituent. The previous studies of Załuski et al. [6] provided that protocatechuic acid has been detected in the 75% ethanol inflorescence extracts from *E. senticosus*, *E. gracilistylus*, and *E. giraldii*, in the amounts of 614.7, 833.4 and 855.6 *μ*g/g DE, respectively. In turn, the 75% ethanol extracts from the fruits of *E. divaricatus* and *E. sessiliflorus* contained 893 and 818 *μ*g/100 g DE of protocatechuic acid. A lower content was quantified in the fruit infusion of these species (270 and 267 *μ*g/100 g DE, respectively) [26].

There are not a lot of reports on phenolic acid content in *Eleutherococcus* species growing in Asia or Russia and in their native habitat. Li et al. [4] studied the content of protocatechuic acid in ten commercial *E. senticosus* samples, purchased from TCM shops of different places in China. The content varied from 3.1 to 23.1 *μ*g/g.

Protocatechuic acid has been determined in many other plants, however, mostly in the aerial parts, which makes it difficult to interpret when compared to underground parts. The aerial parts, usually, contain more phenolic acids.

### 3.3. Antienzymatic Activity

Acetylcholinesterase is the enzyme located in the nervous system and muscles, which regulates the AChE concentration. It decreases the acetylcholine level which implies the gradual loss of memory and learning ability. By far, the AChE inhibitors galantamine, rivastigmine, and donepezil are commonly used. Because of their unfavorable side effects, bioprospecting studies in searching for AChEIs are still ongoing [27–29].

The *Eleutherococcus* spp. are known for their improvement of the mental ability; therefore, the impact of the extracts, at the concentration of 100 *μ*g/0.19 mL of the reaction mixture, on AChE (5 U/mg) activity was measured (Table 5). The five extracts have been tested, for which the inhibition was established to be between 19.6 and 32%, with the highest inhibitory level for *E. gracilistylus* and *E. sessiliflorus* (32%). Physostigmine, used as a control, inhibited AChE at the level of 30% at concentration 2 *μ*g.

The previous studies of Załuski et al. [30] on the anti-AChE activity of the 75% ethanol extracts from the roots of *Eleutherococcus* spp. have shown 50% inhibition for the extract concentration in the range of 300-1750 *μ*g/mL. The highest activity was observed for *E. sessiliflorus* and *E. setchuenensis* (IC_50_ 300 *μ*g/mL), the lowest for *E. henryi* (IC_50_ 1750 *μ*g/mL). It is very interesting that in the case of two types of solvents used, 75% EtOH and 75% MeOH extracts, the same species (*E. sessiliflorus*) has shown the highest inhibitory activity.

Nino et al. [31] studied 27 methanol extracts from different species collected in Colombia (AChE, 0.3 U/mL). *Solanum leucocarpum* Dunal and *Witheringia coccoloboides* (Damm) have shown the highest inhibition (IC_50_ 204.5 and 220.6 mg/L). Nwidu et al. [32] showed that the methanol, ethyl acetate, and aqueous root fractions from *Carpolobia lutea* G. Don inhibited AChE activity with the IC_50_ value 0.3-3 *μ*g/mL. Kostelnik and Pohanka [33] found out that boldine inhibited AChE (100 U/mg) with an IC_50_ value of 372 *μ*mol/L. A new promising group of AChE inhibitors are compounds found in fungi and bacteria. Thabthimsuk et al. [28] studied the anti-AChE activity of polysaccharide-peptide complexes extracted from 3 types of edible fungi, including white shimeji (*Hypsizygus marmoreus*), brown shimeji (*Hypsizygus marmoreus*), and enokitake (*Flammulina velutipes*). The highest activity to inhibit AChE (0.28 U/mL) was showed by brown shimeji (71.3%). In turn, Tan et al. [27] evaluated the anti-AChE (0.22 U) activity of 55 ethyl acetate extracts from 55 bacterial strains belonging to nine bacterial families (Vibrionaceae, Bacillaceae, Microbacteriaceae, Aerococcaceae, Brevibacteriaceae, Staphylococcaceae, Pseudoalteromonadaceae, Enterobacteriaceae, and Shewanellaceae) isolated from coral reefs in Hainan (China). The highest inhibition was shown by *Vibrio neocaledonicus* (98.9%).

In the next step, the impact of the extract on the Hyal (30 U/mg) inhibition, at the concentration 100 *μ*g/0.16 mL of the reaction mixture, was measured (Table 5). An inhibition ranged from 9.1 to 40.7%. The most active was the extract from the roots of *E. henryi* (40.7%). In Załuski et al.'s previous studies, aescin was used as the standard compound. The 30.1% level of inhibition was obtained for the concentration of 100 *μ*g [12]. It should be noticed that the extract possesses chemical structures that may be used as possible inhibitors of Hyal, showing a stronger potency than aescin.

Kuźniewski et al. [34] have shown that the 75% ethanol extracts from the autumn and spring leaves *E. senticosus*, at the concentration of 22 *μ*g/0.16 mL of the reaction mixture, inhibited Hyal at the level of 74.3 and 33%, respectively. The higher inhibition of the autumn leaves might result from the higher amount of polyphenolic compounds, such as tannins.

Furusawa et al. [35] reported the anti-Hyal activity of the coffee silverskin, a by-product of the roasting procedure for coffee beans. The IC_50_ value was at the level of 0.27 mg/mL. In turn, McCook et al. [36] showed that tannic acid, at the concentration of 0.05 mg/mL, inhibited Hyal at the level of 100%. Liyanaarachchi et al. [37] studied the influence of the 18 ethanol extracts on Hyal (4200 U/mL) activity at the concentration of 500 *μ*g/mL. The studies have revealed that only 8 extracts have exhibited such an activity in the range of 34.8 and 95% of inhibition. *Curcuma aromatica* exhibited the best hyaluronidase inhibition activity (95%). Large-scale studies were performed by Hwang et al. [38], in which the inhibitory activity of 500 methanolic extracts of 500 species from medicinal plants was analyzed. Only three MeOH extracts inhibited more than 50% of Hyal activity at a concentration of 2 mg/mL. The level of inhibition for these 3 species, *Styrax japonica* (stem extract), *Deutzia coreana* (stem extract), *Osmanthus insularis* (stem-bark extract), was 57.2, 53.5, and 53.1%, respectively.

Considering the anti-AChE and anti-Hyal activities of different extracts, it is hard to make a reliable comparison because the researchers use the different enzyme activity units and extract concentrations. It is a major problem in antienzymatic activity assays for which the general protocols for the assay should be developed. When the enzyme activity unit was given, we included it in the manuscript. This problem has been resolved, e.g., in case of cytotoxic activity, where according to the National Cancer Institute (United States) plant screening program, a crude extract is generally considered to have *in vitro* cytotoxic activity if the IC_50_ is <20 *μ*g/mL. For compounds, the limit has been established as 4 *μ*g/mL [39].

### 3.4. Cytotoxic Activity

About 6 million of people die of cancer each year, and this trend is about to grow in the next decades. Leukemia is a haematological disease caused by persistence of immature white blood cells in different compartments of the organism mainly the bone marrow, lymph node, spleen, and circulating blood. Plant-based chemicals are a promising group of antileukemic agents, especially the polyphenol group [40, 41]. Because *Eleutherococcus* spp. contain polyphenols, we have studied an antileukemic activity of the extracts. The extracts inhibited a HL-60 cell line growth with an IC_50_ value in the range of 270-2000 *μ*g/mL (Table 6). As a positive control, podophyllotoxin was used, with the IC_50_ value of 0.0084 *μ*g/mL. The previous studies of Załuski et al. [42], on the cytotoxic activity of the 75% ethanol extracts from the roots of *Eleutherococcus* spp., have revealed that the IC_50_ values ranged between 49 and 522 *μ*g/mL, with the highest for *E. divaricatus* (49 *μ*g/mL). Comparing the 75% EtOH extracts to the 75% MeOH extracts, it is seen that both types exhibited a weak inhibitory activity.

The higher IC_50_ values have been obtained for the isolated compounds and their derivatives. Hu et al. [43] studied the cytotoxic activities of five stilbenoids isolated from the stem bark of *Acanthopanax leucorrhizus* and six derivatives against HL-60. Among 17 compounds, only two derivatives (E)-3,5-dimethoxy-3′,4′-methylenedioxystilbene and 2-(3′-hydroxy-4′-isopentenyl-5′-methoxyphenyl)-4-hydroxybenzofuran exhibited moderate cytotoxicities against HL-60 cell lines with the lower IC_50_ values of 30.51 ± 5.37 and 40.23 ± 6.12 *μ*M, respectively. In turn, isolates showed a weak inhibitory activity against HL-60, in the range of 98.6-200 *μ*M.

The cytotoxic activity, against HL-60, was also exhibited by the different extracts [petroleum ether, Ch_2_Cl_2_, EtOAc, EtOH, and EtOH/H_2_O (1 : 1 *v*/*v*)] prepared from the aerial parts of *Artemisia biennis* Willd. Nevertheless, high IC_50_ values have been obtained, in the range of 54.3-200 *μ*g/mL [44]. The *n*-hexane, Ch_2_Cl_2_, EtOAc, EtOH, and EtOH/H_2_O (1 : 1 *v*/*v*) extracts from another Artemisia species, *Artemisia turanica* Krasch., have also shown a weak cytotoxic effect with the IC_50_ values of 68.8-450 *μ*g/mL [45]. It should be mentioned that petroleum ether, dichloromethane, ethyl acetate, ethanol, and 50% aqueous ethanol extracts of the aerial parts of *Artemisia ciniformis* inhibited HL60 cell growth with the IC_50_ value of 31.3-200 *μ*g/mL [46].

Teixeira et al. [47] reported on the cytotoxic activity of four flavonoids isolated from the roots of *Tephrosia egregia* Sandwith. Three compounds (pongaflavone, praecansone B, and 12a-hydroxyrotenone) exhibited a high activity against HL-60 cell line with the IC_50_ values of 1.4, 8.1, and 1.9 *μ*g/mL.

Taking into account the antileukemic activity of polyphenols, we noticed that *E. henryi* was the most active towards HL-60 and contains the highest amount of protocatechuic, *p*-coumaric, and caffeic acids. Another observation resulted from this study is that nonpolar extracts are more cytotoxic than polar ones. A moderate/low cytotoxicity in our study may result from a high polarity of extract constituents, for which a cell wall penetration is limited. Further studies are needed to confirm this activity for the isolated compounds.

### 3.5. The Capacity of DPPH^∗^ Reduction

**Figure 2:**
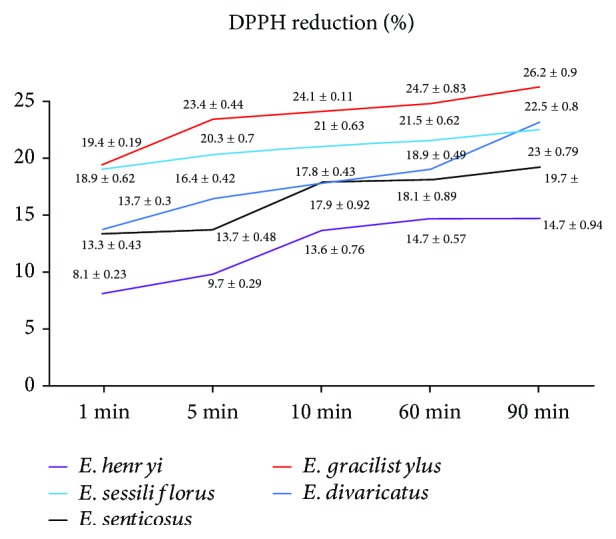
A time-dependent DPPH^∗^ reduction (%).

It is known that free radicals are responsible for the development of many diseases, such as Parkinson's disease, Alzheimer's disease, or amyotrophic latera sclerosis. Free radicals are constantly produced in the brain *in vivo*, contributing to oxidative damage of proteins and lipids. For this reason, in searching for new antioxidants, a special attention should be paid to compounds that act as an antioxidant and inhibitor of AChE or BuChE. Nature is a significant source of health-promoting compounds that may be divided into two groups: fruits and vegetables and medicinal plants [48]. Therefore, in the next step, an antioxidative activity of the extracts was examined. The extracts reduced DPPH^∗^ in a time-dependent mode, at the concentration of 0.8 mg/mL (Figure 2). After 90 min from 14.7 to 26.2%, DPPH^∗^was reduced. As a positive control, ascorbic acid was used, with an EC_50_ value of 38 *μ*g/mL (after 5 min of incubation). A slow change of antioxidant activity in the time is in agreement with the adaptogen's definition, according to which, adaptogen should act slowly in a long-term mode. It should be noticed that *E. gracilistylus* expressed the highest anti-AChE activity and anti-DPPH^∗^ scavenging. Usually, free radicals are associated with the neurodegenerative process development; in this case, we can suppose that *E. gracilistylus* contain both compounds with anti-AChE activity and anti-DPPH^∗^.

Since a few years, the main attention has been paid to fungi as a new source of antioxidants. By far, they were only of culinary interest; however, around 650 species contain therapeutic properties, e.g., *Lentinus edodes*, *Boletus edulis*, or *Grifola frondosa*. They are rich in phenolic acids, such as cinnamic, protocatechuic, *p*-hydroxybenzoic, *p*-coumaric, and gallic [48].

The acetonic extracts from *Boletus edulis* and *Boletus aestivalis* have showed a powerful antioxidant activity with the IC_50_ value of 4.72 and 8.63 *μ*g/mL which were similar or greater than those of the standard antioxidants, ascorbic acid (EC_50_ 4.22 *μ*g/mL), BHA (EC_50_ 6.42 *μ*g/mL), and *α*-tocopherol (EC_50_ 62.43 *μ*g/mL) [49]. Higher values have been obtained for *Agaricus brasiliensis* and *Agaricus bisporus*. The 60% ethanol extracts from those fungi have shown a radical scavenging capability for EC_50_ 1.6 and 2.7 mg/mL. In turn, the aqueous extracts have had the EC_50_ value at the level of 3.8 and 4.5 mg/mL [50].

## 4. Conclusions

The ethnopharmacological uses of *Eleutherococcus* spp. are now confirmed with an application of new approaches and tools, in *in vitro* and *in vivo* models. Considering the inhibition of an AChE activity and DPPH^∗^ reduction, new inhibitors should be searched for in the roots of *E. gracilistylus*, while inhibitors with an anti-Hyal or cytotoxic activity should be searched for in the roots of *E. henryi*. New Hyal inhibitors may have a possible use as ingredients of plant-based products, used, e.g., topically as cosmetics or in treatment of skin diseases. Taking into account the results obtained in this work, a further research should focus on the isolation of single phytochemicals being responsible for the anti-AChE, anti-Hyal, and cytotoxic effects. Further researches are needed to determine the antienzymatic activity with the use of modern approaches, such as the isothermal calorimetric titration (ITC).

## Figures and Tables

**Table 1:** Yield, TPC, and TFC in 75% methanol extracts from the roots of *Eleutherococcus* spp. (g GAE/g and QEs/g DW^∗^).

Sample	Yield (%)	TPC	TFC
*E. gracilistylus*	10.7	4.1 ± 1.4	1.8 ± 0.02
*E. divaricatus*	5.7	9.4 ± 0.9	6.5 ± 1.1
*E. senticosus*	8.3	7.9 ± 0.3	4.6 ± 0.9
*E. henryi*	6.4	10.4 ± 1.3	4.8 ± 0.3
*E. sessiliflorus*	10.7	9.7 ± 0.5	6.2 ± 0.7

^∗^Results are the means ± standard deviation of triplicates.

**Table 2:** Optimized LC-MS (MRM) parameters for all analytes. Compounds confirmed by comparison with authentic standards.

Compound	Retention time (min)	Q1 (*m*/*z*)	Q3 (*m*/*z*)	DP^a^ (V)	EP^b^ (V)	CEP^c^ (V)	CE^d^ (eV)	CXP^e^ (eV)
Gallic acid	1.01	168.7	178.9 124.9	-35 -35	-3 -3	-12 -12	-36 -14	0 0
Protocatechuic acid	2.16	152.9	80.9 107.8	-55 -55	-1 -1	-10 -10	-26 -38	0 0
Gentisic acid	3.32	352.9	80 96.9	-70 -70	-4 -4	-16 -16	-110 -52	0 0
4-OH-benzoic acid	4.25	136.8	92.9	-30	-7	-10	-18	0
Vanillic acid	5.51	166.8	107.9 123	-35 -35	-4 -4	-12 -12	-18 -12	0 0
Caffeic acid	5.69	178.7	88.9 134.9	-30 -30	-6.5 -6.5	-12 -12	-46 -16	0 0
Syringic acid	6.34	196.9	122.8 181.9	-30 -30	-9 -9	-12 -12	-24 -12	0 -2
*p*-Coumaric acid	6.67	162.8	93 119	-30 -30	-8 -8	-12 -12	-44 -14	0 0
Ferulic acid	6.83	192.8	133.9 177.9	-25 -25	-11.5 -11.5	-14 -14	-16 -12	0 -2
Salicylic acid	6.86	136.9	75 93	-35 -35	-4 -4	-10 -10	-48 -16	0 -2
Veratric acid	6.88	180.7	121.9 136.9	-35 -35	-6 -6	-14 -14	-18 -12	0 0
Sinapic acid	6.88	222.8	121 148.9	-35 -35	-8.5 -8.5	-10 -10	-36 -20	0 0
*m*-Coumaric acid	6.89	162.8	91 119	-35 -35	-4.5 -4.5	-12 -12	-36 -14	0 0
Rosmarinic acid	7.01	358.7	132.6 160.8	-50 -50	-5 -5	-26 -26	-44 -20	0 -2

^a^DP: declustering potential; ^b^EP: entrance potential; ^c^CEP: cell entrance potential; ^d^CE: collision energy; ^e^CXP: collision cell exit potential.

**Table 3:** Limit of detection (LOD), limit of quantification (LOQ), and calibration curve parameters for all analytes.

Compound	LOD (ng/*μ*L)	LOQ (ng/*μ*L)	*R*^2^	Linearity range (ng/*μ*L)
Gallic acid	0.05	0.1	0.9991	0.1- 10
Protocatechuic acid	0.01	0.02	0.9967	0.025-25
Gentisic acid	0.008	0.015	0.9993	0.025-25
4-OH-benzoic acid	0.05	0.1	0.9972	0.1-5
Vanillic acid	0.1	0.2	0.9999	0.2-50
Caffeic acid	0.04	0.085	0.9975	0.1-5
Syringic acid	0.05	0.1	0.9997	0.1-50
*p*-Coumaric acid	0.01	0.025	0.9982	0.05-2.5
Ferulic acid	0.01	0.025	0.9997	0.025-5
Salicylic acid	0.01	0.02	0.9986	0.02-0.7
Veratric acid	0.4	0.7	0.9977	0.5-25
Sinapic acid	0.007	0.025	0.9987	0.025-5
*m*-Coumaric acid	0.02	0.05	0.9994	0.05-2.5
Rosmarinic acid	0.005	0.01	0.9985	0.025-25

**Table 4:** Phenolic acid contents expressed in *μ*g per 1 g of dry weight of extracts. Mean values of three replicate assays with standard deviation.

Phenolic acid	*E. gracilistylus*	*E. divaricatus*^∗^	*E. senticosus*^∗^	*E. henryi*^∗^	*E. sessiliflorus*^∗^
Protocatechuic acid	446 ± 1	435.5 ± 5.5	838 ± 12	1865 ± 35	529 ± 1
Salicylic acid	<LOQ	30.6 ± 0.2	16.8 ± 1	<LOQ	41.4 ± 2.6
Caffeic acid	nd	49 ± 0.3	135.5 ± 3.5	244 ± 2	49.1 ± 0.3
*p*-Coumaric acid	30.6 ± 0.95	12.35 ± 0.12	23.6 ± 0.3	55 ± 0.2	<LOQ
Ferulic acid	64.6 ± 3	46.6 ± 3	43.9 ± 2.4	55 ± 1.8	11 ± 0.3

nd: not detected.

**Table 5:** Antihyaluronidase and antiacetylcholinesterase activities of extracts.

Sample	Anti-AChE^∗^	Anti-Hyal^∗^
	(% inhibition)	
*E. gracilistylus*	32 ± 0.8	14.9 ± 0.3
*E. divaricatus*	23.2 ± 0.9	11 ± 0.2
*E. senticosus*	26.1 ± 0.05	10.4 ± 0.6
*E. henryi*	19.6 ± 0.4	40.7 ± 1.1
*E. sessiliflorus*	32 ± 0.6	9.1 ± 0.05

^∗^Results are the means ± standard deviation of triplicates.

**Table 6:** The cytotoxic impact of the extracts on HL-60 cell line (IC_50_ *μ*g/mL).

*E. gracilistylus*	*E. divaricatus*^∗^	*E. senticosus*^∗^	*E. henryi*^∗^	*E. sessiliflorus*^∗^
890 ± 3.7	650 ± 2.9	450 ± 2.5	270 ± 1.1	2000 ± 3.4

## Data Availability

The data used to support the findings of this study are available from the corresponding author upon request.

## Abbreviations

TPC: Total phenolic content
TFC: Total flavonoid content
DNPH: 2,4-Dinitrophenylhydrazine
FeCl_3_: Iron (III) chloride
GA: Gallic acid
HE: Hesperetin equivalent
QE: Quercetin equivalent
DPPH^∗^: 2,2-Diphenyl-1-picrylhydrazyl
HL-60: Human Caucasian promyelocytic leukemia from American Type Culture Collection (ATCC CCL-240™)
Hyal: Hyaluronidase
AChE: Acetylcholinesterase.
